# The Effects of Music during a Physical Examination Skills Practice: A Pilot Study

**DOI:** 10.3390/vetsci4040048

**Published:** 2017-09-27

**Authors:** Elpida Artemiou, Gregory E. Gilbert, Fortune Sithole, Liza S. Koster

**Affiliations:** 1 Department of Clinical Sciences, Ross University School of Veterinary Medicine, P.O. Box 334 Basseterre, St. Kitts, West Indies; FSithole@rossvet.edu.kn; 2 Learning Sciences, Adtalem Global Education, 3005 Highland Parkway, Downers Grove, IL 60515, USA; GGilbert@rossu.edu; 3 Center for Teaching and Learning, Ross University School of Medicine, P.O. Box 266 Roseau, Commonwealth of Dominica, West Indies; 4 Small Animal Hospital, University of Glasgow Veterinary School, 464 Bearsden Road, Bearsden G61 1QH, UK; liza.koster@glasgow.ac.uk

**Keywords:** music, psychoacoustic music, stress, mood and arousal, veterinary medical education

## Abstract

Some veterinary students experience elevated stress, anxiety, and depression resulting in disease and psychological changes. Elevated arousal, negative moods, and lack of interest can negatively affect performance and learning. Psychoacoustic music promotes calming effects using simple and slow piano sounds and can positively impact well-being and functioning. This pilot study assessed the effects of music on blood pressure, pulse, arousal, and mood during a canine physical examination laboratory. In an AB/BA crossover study, 17 students were randomly allocated to practice physical examination skills while listening to *Through a Dog’s Ear, Volume 1*. Psychological and physiologic data were collected. Nonparametric methods were used to test for significant differences in psychological and physiologic data and a linear mixed models approach was used to test for physiological differences. There were no significant baseline differences between the music and no music groups for DASS-21 depression, anxiety, or stress scores; however, there were significant time differences between pretest and posttest on arousal and mood as measured by the Profile of Mood Sates (POMS) Depression, Fatigue–Inertia, and Tension Anxiety subscales. Linear mixed models revealed no significant treatment effect on the pulse and diastolic blood pressure; however, there was a significant systolic blood pressure treatment effect. Future indications include repeating the study with a larger sample to examine longitudinal psychological and physiological benefits.

## 1. Introduction

A significant number of medical and veterinary students experience various levels of psychological distress and depression during training [1,2]. Although psychological distress in medical and veterinary students has been examined thoroughly, further evidence is required surrounding curricular changes and learning environments promoting positive emotional, physical and academic well-being [2,3,4]. Similar to medical students, veterinary students undergo challenges related to living away from home, experience general health issues, and are faced with challenging peer interactions, all of which can result in physiological changes and disease [3,4,5,6,7]. In addition, the demanding academic workload and frequent assessments are contributing factors to students’ psychological distress [8]. One recent study estimated that 30% of veterinary students were at risk of burnout, while 21% reported low compassion satisfaction levels [9]. Psychological distress can affect students physiologically, and academic performance can also suffer [2,7]. When students experience psychological distress, sustained attention and working memory are allocated to management of symptoms related to stress, anxiety, and depression rather than educational pursuits [4,7,8,9,10]. This shunting of focus and effort toward their psychological state may distract from and decrease academic performance [4,7,8,9,10].

It has been shown that psychological distress, general health, and over-the-counter (OTC) medication are linked with a negative impact on student grades [4,11]. Kogan and associates (2005) examined a number of non-academic stressors in veterinary students [12] and showed that few students are really conscious of the influence of stress on their performance, and there is a large gender influence, with females reporting having an overwhelmed feeling 6 to 10 times or more than 10 times in the past year [12].

The opportunity exists to further explore how auditory stimulation can support teaching and learning and promote students’ well-being. Auditory stimulation involves enriching an environment using music and has been demonstrated to positively impact a person’s well-being and functioning [13]. Music therapy dates to 6000 BCE when it was played during healing ceremonies [14]. In modern times, music was first used post World War II to treat wounded soldiers and raise their morale [14]. The therapeutic physiological and psychological outcomes for humans have been well documented [13,14,15].

To our knowledge, limited work exists evaluating the use of music in medical education [16]. One example demonstrated when music was played during a pharmacology class it was found to enhance student recall [17]. Studies on humans show music positively influences mood and emotions, enhances prosocial behaviors, and has therapeutic effects on preoperative anxiety and pain, and improves cognition and spatial ability [13,18]. The influence of music on performance can be potentially explained through the arousal-mood hypothesis, which postulates that certain types of music may lower negative moods and may lower arousal associated with anxiety [15,19]. Evidence supports that anxiety, arousal, negative moods states, or lack of interest can negatively affect performance and learning [19,20,21]. Particular interest involves the use of psychoacoustic music. Psychoacoustic music refers to musical arrangements following psychoacoustic principles such as playing slower and simple piano sounds, and the use of individual instruments versus complex orchestral sounds to promote calming effects and reduce anxiety behaviors [22]. The Profile of Mood States (POMS) questionnaire allows for an assessment of changes in individuals’ states of arousal and mood prior to and after listening to music [15,19]. Evidence links the POMS Depression–Dejection with feelings of increased self-blame and hopelessness while Tension–Anxiety refers to worry and anxiety, and Fatigue–Inertia are associated with experiencing low mental and physical energy [15,23]. Construct validity demonstrates that the POMS Depression–Dejection subscale is associated with changes in mood, as well as correlates with other questionnaires such as the Beck Depression Inventory [15]. Vigor–Activity describes feelings of an active, lively, and vigorous presence and has high reliability and links with arousal [15], and the negative measure of Tension–Anxiety subscale can be a potential measure of arousal associated with anxiety. The Fatigue–Inertia subscale can potentially link with low arousal and/or mood [15]. Associated physiological responses to music can also include improvements in blood pressure, pulse, and salivary immunoglobulin IgA [24,25].

Although some evidence of music therapy exists in medical education, studies examining the use of music in veterinary medical education could not be found. The teaching and learning of veterinary medicine requires the use of animals, a practice that students can find rewarding and comforting; however, from an animal perspective, this can pose challenges surrounding animal welfare concerns and the need to assess the physiological impact of repeatedly practicing clinical skills on teaching colonies [26]. To this end, our pilot study assessed the effect of music on both canine subjects and veterinary students with a minimum of one year of veterinary training during a canine physical examination skills laboratory. We chose to use animal patients instead of simulators as it replicated a physical examination teaching laboratory, as well as provided a more realistic representation of veterinary practice. The focus of this manuscript is on the effects of music on veterinary students. Because of the proven effects of music, we decided to investigate, in this preliminary study, whether having music during a physical examination laboratory could have a positive effect on student stress during animal handling. We postulated practicing physical examination skills, while listening to music, would result in reduced blood pressure, pulse, arousal, and improve mood in veterinary students. Our study results can further inform the evidence surrounding music in veterinary medical education, as well as support changes enhancing teaching and learning laboratory settings.

## 2. Materials and Methods

### 2.1. Participants

In the Fall of 2015, members of the Student Animal Behavioral Club of a large offshore AVMA-accredited veterinary medical school were invited to participate in this study. Participants were selected if they had successfully completed the physical examination laboratory offered at the end of year one of the curriculum. All participants provided informed consent. The study was approved by the institution’s Institutional Review Board (IRB) and was conducted in accordance with the tenets espoused in the Declaration of Helsinki (approval code: EA15-07-R1).

### 2.2. Study Design and Data Collection

We used a randomized AB/BA crossover design in which the same participants received the treatment (physical examination skills with music), and control (physical examination without music). This allowed us to reduce the variability of the multiple measurements on a participant, as well as a more precise treatment comparisons and control of confounding variables. The participants acted as their own controls, and in our case since the only treatment factor was the intervention of music, we were able to control for the known effect of animal interaction as a stress reliever, thus being able to measure the true effect of the intervention-music, and best minimize the presence of this confounder.

Immediately prior to both laboratory practice sessions, the students completed the short-form of the Depression Anxiety Stress Scales (DASS-21) [27,28] to provide a baseline self-assessment of depression, anxiety, and stress. In addition, before and after the practice session we evaluated (i) psychological changes using the Profile of Mood States (POMS) inventory [29], and (ii) physiological changes were assessed through measurements of blood pressure and pulse rate. Students’ blood pressure and pulse were recorded by the university nurse using standard methods immediately after completion of DASS and POMS questionnaires, and prior to the beginning of the practice session. Both pressures and pulse were taken while in the seated position. During the practice session, students were supported by a small animal specialist (LK) that provided guidance and assistance as well as confirmation that all participants successfully practiced and completed all required clinical tasks. As students completed their physical examination skills, the nurse was called to their station and took the second readings. There were no delays in taking either of these physiological measurements.

The students participated in a 60-min laboratory to practice physical examination skills while listening to music (*Through a Dog’s Ear, Volume 1*) [22] or were in an adjacent room practicing without music. Volume 1 includes nine soundtracks with a total running time of 59:58 min, designed to induce calming effects for both canines and humans [22,30,31]. Music was played at a range of 55 ± 5 dB, 1–6 m from students. After a seven-day washout period, the experiment was repeated and participants were crossed over based on their previous random allocation to practicing skills with or without music.

### 2.3. Statistical Analysis

Data were entered in Microsoft Excel^®^ 2016 (Redmond, WA, USA) software and analyzed using R v3.3.1 (Vienna, Austria). Data were assessed for normality and nonparametric methodology was used to describe the data. DASS-21 and POMS scores were tested for normality using normal probability plots, the Anderson-Darling, Shapiro-Francia, and the Shapiro-Wilk normality tests [32,33]. Goodness-of-fit testing used an a priori alpha level of 0.05.

Median and interquartile range were used to describe the data and Wilcoxon-Mann-Whitney and Wilcoxon rank sum tests were used for significance testing, as appropriate for independent and paired data. A random intercept, random slopes model was used to assess heart function parameters. As this was an education intervention, an a priori alpha level of 0.10 was specified [34].

## 3. Results

Seventeen of 45 (38%) rising second-year veterinary students in the Student Animal Behavior Club volunteered for this study and met the study criteria. The investigation took place between November 2015 and February 2016.

### 3.1. Normality Testing

The DASS-21 and POMS pretest scores were tested for normality using normal probability plots and the Anderson-Darling, Shapiro-Francia, and the Shapiro-Wilk normality tests [32,33]. All DASS-21 subscales were judged to be not normally distributed. Six of the nine POMS subscales were judged to be not normally distributed. In the interest of ease of presentation and interpretation, the decision was made to describe all the data using median and interquartile range (IQR) and to test for significant difference using nonparametric methods (Wilcoxon-Mann-Whitney test and the Wilcoxon signed rank test).

### 3.2. DASS-21

Median values for the depression subscale of the DASS-21 for the control group were 2 (IQR: 7.5) as compared to the intervention group (median: 3; IQR: 6.5), and both considered within normal scores for depression relative to the general population. Median stress scores were identical for intervention and control groups (median: 5 (IQR: 5.0) and median 6 (IQR: 10.5), respectively), and both considered within normal scores for stress relative to the general population. Median anxiety scores were identical for intervention and control groups (median: 5 (IQR: 5.0) and median 6 (IQR: 10.5), respectively), indicating that participants in the intervention group experienced mild symptoms of anxiety, while participants in the control group reported moderate symptoms of anxiety relative to the population. Wilcoxon-Mann-Whitney tests revealed no significant differences between depression scores (*p*-value = 0.4628), anxiety scores (*p*-value = 0.6604), or stress scores (*p*-value = 0.3828).

### 3.3. POMS

The POMS results are summarized in Table 1. Median values for the Depression–Dejection subscale of the POMS were similar for the intervention and control groups. There was no significant treatment effect difference for the Depression–Dejection subscale, although there was a significant difference (*p*-value = 0.0082) between the pretest and posttest scores independent of treatment effect.

Median values for the Fatigue–Inertia subscale of the POMS with music were higher for the pretest as compared to the posttest group. There was a significant difference (*p*-value = 0.0002) between the pretest and posttest scores.

Differences in the Tension–Anxiety subscale of the POMS showed the largest pretest and posttest differences. There was no significant difference for the Tension–Anxiety subscale, although as with other subscales there was a significant time effect difference (*p*-value = 0.0001) between the pretest and posttest scores independent of the treatment effect.

### 3.4. Physiological Parameters

A summary of physiological parameters (pulse, systolic blood pressure, and diastolic blood pressure) can be seen in Table 2. Medians and interquartile ranges are reported along with *p*-values for a Wilcoxon signed rank test testing. Statistically significant decreases were measured in pulse and systolic blood pressure in the intervention and control groups. A clinically important difference (11 bpm) in median pulse rate was seen in the intervention group and about half the difference was seen in the median pulse rate in the control group (5 bpm). A smaller decrease was measured in systolic blood pressure in both groups, which can be considered of less clinical importance. There were no differences in diastolic blood pressure.

There was no significant treatment effect on the pulse (*p*-value = 0.2369); however, there was a significant difference between pretest and posttest values (*p*-value = 0.0010). There was a significant difference in systolic blood pressure between the treatment and intervention groups (*p*-value = 0.0341) and, as with the pulse data, there were significant differences between the pretest and posttest groups (*p*-value = 0.0081). There were no significant differences between treatment and intervention groups (*p*-value = 0.2144) for diastolic blood pressure, and there were no significant differences between pretest and posttest values with respect to diastolic blood pressure (*p*-value = 0.3062).

### 3.5. Effect Size

Effect sizes were calculated for POMS results. Medium effect sizes were seen for the POMS Depression–Dejection and Fatigue–Inertia subscales (0.5 and 0.4, respectively), and were interpreted using McGraw and Wong’s Common Language Effect Size statistic (CLES). A large effect size (0.9) was seen for the POMS Tension–Anxiety subscale. We can interpret this to mean that 82 out of 100 veterinary students had a higher Tension–Anxiety subscale score during the pretest as opposed to the posttest [35].

## 4. Discussion

This preliminary study assessed the effect of music on rising sophomore veterinary students during a canine physical examination skills laboratory. Using an AB/BA study design, we accounted for individual differences and some confounding variables. Music is known to influence mood with the range in enjoyment levels characterized from low arousal and sad mood to high arousal and positive mood [15,19]. By introducing music during a routine physical examination of a dog, it was our hope to positively influence student mood and arousal and physiological responses.

Prior to physical examination practice session, students completed the DASS-21—an instrument with demonstrated validity and reliability for assessing depression, anxiety, and stress [27,28]. The DASS-21 includes 21 statements that responders rate using a four-point scale as follows: 0 = did not apply to me at all (never) over the past week, 1 = applied to me to some degree, or some of the time (sometimes) over the past week, 2 = applied to me a considerable degree, or a good part of time (often) over the past week, and 3 = applied to me very much, or most of the time (almost always) over the past week. The aggregate DASS-21 results indicated that only the anxiety scale could have negatively contributed to the overall well-being and performance of the participants, as the intervention and control groups respectively indicated mild and moderate anxiety baseline symptoms compared to the general population. Establishing this baseline surrounding depression, anxiety and stress through DASS-21 precludes high emotional participant distress and significant impact on clinical performance.

Additionally, pre-post physical examination practice sessions, students completed the POMS—an inventory that is a psychometrically sound self-report instrument, designed for adults, used to record an individual’s mood state [29]. The POMS includes 65 adjectives describing feelings and mood that responders rate based on a five-point Likert scale ranging from 0 = not at all, 1 = a little, 2 = moderately, 3 = quite a bit, to 4 = extremely. POMS responses are presented on six subscales: Anger, Confusion–Bewilderment, Depression–Dejection, Fatigue–Inertia, Tension–Anxiety, and Vigor–Activity. Participants completed the entire POMS instrument ensuring the instrument’s psychometric properties remained intact, though we were interested in one scale reflecting participants’ mood: Depression–Dejection, and two scales describing arousal levels: Tension–Anxiety and Fatigue–Inertia.

Specifically, the POMS results indicate that the music did not significantly lower negative affect, nor did it improve students’ mood, as measured by the Depression–Dejection POMS scale. Likewise, listening to music did not result in a lowering of negative arousal as assessed by Tension–Anxiety and Fatigue–Inertia POMS scales. These results are not unexpected considering the reported aggregate baseline DASS-21 results, though not consistent with evidence linking music with strong intentional emotional responses affecting both arousal and mood as well as potentially decreasing anxiety [36,37]. We expected that the intended emotional response of participants experiencing music to report a higher expression of emotions such as being happy and energized. However, we must recognize that tempo (how fast or slow the music is played) and mode (whether music played in major or minor key) correlate with arousal and mood, respectively, and that the ideal tempo varies for major and minor modes and how they influence individuals to experience enjoyment [15], which also contributes to the complexity of physiological responses [38].

Regardless of treatment effect, music or no music, both groups showed a significant time effect (*p*-value < 0.01). In other words, there was a significant reduction on the POMS scales of Depression–Dejection, Tension–Anxiety, and Fatigue–Inertia between pretest and posttest. One explanation for these results is that the mere completion of the required tasks at the end of the practice session may have contributed to significantly reported reduced levels particularly for the Tension–Anxiety subscales at the end of the exercise. Additionally, repetition of the physical examination skills can improve confidence and reduce anxiety.

Participants performing the physical examination with music noted a significant decrease in systolic blood pressure. This result is in agreement with previous research findings that compared pre-post results with stress stimuli and demonstrated music to be an effective approach to managing anxiety and related physiological responses [21]. Participants not experiencing music showed significant time affect changes in systolic and pulse rate, findings that are supported by previous research [39,40] indicating that physical interaction of stroking an animal, and potential familiarity with the dog, can result in transient changes in pressure and heart rate. Further attention needs to be paid to this confounding factor and the potential physiological changes experienced.

One of the major limitations of this study is that the music chosen may also have an effect on the behavior of the canine subjects and not only on the human subjects. Indeed, this music has been shown to have positive effects on human and canine subjects [30,31]. We do recognize the confounding variable of the music may have reduced canine anxiety and stress, making it easier to complete the exercise, thus reducing stress in students in the intervention group.

Another important limitation is the presence of a live dog and the positive association between human–animal interaction that needs to be further evaluated, as it is a highly diverse variable, and although the study design is appropriate in best managing this confounder, our small sample size cannot completely exclude the human–animal variability as a random one.

A further limitation relates to the DASS-21 self-report pre-screening tool results. The DASS-21 results indicated no significant differences between the groups experiencing music while examining the dogs and participants not experiencing music; however, student participants in both groups might be clinically diagnosed with depression, stress, and/or anxiety, and as such could potentially be receiving psychotherapy and/or medications; we did not control for existing depression or treatment of depression. Additionally, a self-reported assessment of mood often poses both a difficulty in interpretation, as well as a challenge in the usefulness of the data with regard to reliability and validity [41]. One way our study is significant is that it exposes the challenges that exist when assessing the effects of music surrounding emotions—an understanding that is extremely useful in designing future investigations.

Another possible explanation for not finding a significant reduction in stress was that, perhaps, students were not very stressed during the lab. If students were not highly stressed, it would make it much harder to find an effect of stress and anxiety reduction. One way to better investigate this topic may be to measure the stress and anxiety of students going into two high stakes exams known to be stressful and introduce music during one of the exams.

Lastly, the lack of significant findings may be attributed to an underpowered study. Any future studies should include an a priori power analysis to determine an appropriate sample size. The fact that students were volunteers and potentially interested in the study also poses a bias, and that a true randomized participant sample described by additional characteristics such as gender, age, and other characteristics may be of particular interest. However, this investigation was framed as a pilot study and as there appears to be a paucity of literature regarding the use of music therapy in reducing stress in students of veterinary medicine, we believe this investigation makes a substantial contribution to the literature.

## 5. Conclusions and Future Directions

In this preliminary study, we showed that the reported scores on the Depression, Fatigue–Inertia, and Tension–Anxiety Profile of Mood States subscales were lower at the end of the exercise, though not as a result of the introduction of music therapy. Music therapy decreased heart rate significantly in veterinary students. We can thus conclude that future studies should thus include a larger group of students and assess the effect of music while students practice clinical skills other than a physical examination. Different types of music and simulated environments should also be included.

## Figures and Tables

**Table 1:** Pretest and posttest median, interquartile range, and *p*-values for the Wilcoxon signed rank test for Depression–Dejection, Fatigue–Inertia, and Tension–Anxiety POMS subscales.

POMS Subscale	Music		No Music		*p* Value
	Pretest	Posttest	Pretest	Posttest	
	Median (IQR)	Median (IQR)	Median (IQR)	Median (IQR)	
Depression–Dejection	1 (3.25)	0 (1)	0 (4)	0 (1)	0.2948
Fatigue–Inertia	7 (5.25)	4 (5)	7 (6)	6 (4)	0.2293
Tension–Anxiety	9 (8)	3 (4.5)	7 (6)	5 (4)	0.9154

IQR—Interquartile range.

**Table 2:** Median and interquartile range for pulse, systolic blood pressure, and diastolic blood pressure (and *p*-value for Wilcoxon signed rank test) for participants under intervention and no music conditions.

	Music		*p* Value	No Music		*p* Value
	Pretest	Posttest		Pretest	Posttest	
	Median (IQR)	Median (IQR)		Median (IQR)	Median (IQR)	
Pulse	85 (14.5)	74 (22)	<0.0001	83 (18)	78 (16)	0.0207
SBP (mmHg)	117 (15)	114 (16)	0.0963	120 (13)	116 (18)	0.0506
DBP (mmHg)	76 (8.5)	76 (7.5)	0.3984	77 (9.0)	75 (9.0)	0.2633
